# The influence of sagittal profile alteration and final lordosis on the clinical outcome of cervical spondylotic myelopathy. A Delta-Omega-analysis

**DOI:** 10.1371/journal.pone.0174527

**Published:** 2017-04-21

**Authors:** Daniel Koeppen, Claudia Piepenbrock, Stefan Kroppenstedt, Mario Čabraja

**Affiliations:** 1 Department of Orthopedic Surgery and Traumatology, Bundeswehrkrankenhaus Berlin, Berlin, Germany; 2 Joint Spine Centre, Vivantes Auguste Viktoria Klinikum, Berlin, Germany; 3 Department of Orthopedic Surgery, Sana Kliniken Sommerfeld, Kremmen, Germany; University of Toronto, CANADA

## Abstract

**Purpose:**

Decompression and maintaining or restoring a cervical lordosis are major goals in the surgical treatment of cervical spondylotic myelopathy (CSM). Numerous studies support the assumption that cervical lordosis is a key factor for neurological recovery and pain reduction. However, even kyphotic patients can be asymptomatic. The balance of the spine is subject of an increasing number of publications. The main purpose of the study was to evaluate the validity of lordotic alignment on the course of CSM and to set this parameter in context with well-validated tools, namely the modified Japanese Orthopaedic Association scoring system (mJOAS) and the visual analogue scale (VAS), to predict and measure the clinical outcome after surgery.

**Methods:**

This is a retrospective study with prospectively collected data of a heterogeneous cohort. The authors analyzed the records of 102 patients suffering from CSM that underwent decompressive surgery and instrumentation. Clinical outcome was assessed by using the mJOAS, VAS and Odom’s criteria. The radiological analysis involved comparison of pre- and postoperative radiographs. The patients were divided into subgroups to be able to compare the influence of various amounts of correction (3 Delta-groups: <0°, 1–7° and ≥8°) and final lordosis (4 Omega-groups: 0–7°, 8–14°, 15–21°, ≥22°).

**Results:**

219 levels were fused in 102 patients. Surgery improved the clinical outcome of all groups significantly. A lordotic profile was achieved in all analyzed groups. Patients that showed small lordosis after surgery (<8°) did not have an inferior clinical outcome compared to patients with larger cervical lordosis (>14°). The comparison of Odom’s criteria showed that preoperatively kyphotic patients benefitted more from surgery than lordotic patients (p = 0.029), but no differences could be seen comparing neck pain and neurological improvement. The improvement of pain and neurological impairment measured by VAS and mJOAS supports the statistical impact and validity of the data despite comparatively small numbers of patients. The lack of postoperative kyphosis is a major limitation of the study to encompass the impact of sagittal alignment on clinical outcome.

**Conclusions:**

Decompression and stabilization appear to be key elements of surgical treatment of CSM. While the achievement of cervical lordosis remains a major goal of surgery, clinical improvement is not hindered in patients who show small lordosis. However, kyphosis should be eliminated in symptomatic patients. The terms “balance” and “physiologic lordosis” remain complex entities without clear definition. To check the results of our study controlled randomized trials to validate and determine the exact role of cervical balance on the course of CSM would be helpful.

## Introduction

Cervical spondylotic myelopathy (CSM) is a common cause of neurological morbidity and can be effectively treated by surgery [1–4]. Decompression of the spinal cord and restoration or maintenance of lordotic cervical alignment are accepted surgical treatments of CSM [5–7].

Psychological comorbidities, age of patients, baseline modified Japanese Orthopaedic Association score (mJOAS), stability of gait and duration of symptoms appear to be key factors for clinical outcome [8–10]. Furthermore cervical sagittal balance before and after surgery appears to have a major influence on the clinical course of patients [1, 11–15]. Cervical alignment can be measured locally by Cobb angle, which is still the most commonly used local measurement method due to its validity. C2-7 lordosis changes with age, but typically ranges from 15–25° [5].

Severe cervical deformity needs to focus on global balance as well. Translation of the cervical spine in the sagittal plane is measured through the cervical sagittal vertical axis (SVA) and is ideally defined as a C7 plumb line less than 5 cm [16]. The SVA can be locally used by measuring the distance between a plumb line from C2 and C7 and is reported to be 16.8 ± 11.2 mm in asymptomatic patients [17]. More complex measurement concepts include the neck tilt, T1 slope and thoracic outlet angle to determine the physiological cervical alignment, but the data is controversial in parts [18, 19].

Clinical outcome appears to be influenced by a reduced C2-7 lordosis or larger C2-7 SVA [5, 11, 12, 20, 21]. Nonetheless, numerous studies also exist that do not see any clear correlation between clinical outcome and cervical lordosis [2, 22, 23]. Furthermore, patients with a severe kyphosis can often not be corrected into a physiological lordosis but still profit substantially from surgery [24]. The spinal cord’s shift resulting from a large correction might bear disadvantages such as a C5 palsy as well [25]. However, even kyphotic patients are often asymptomatic [26]. This complicates the terminology of a physiologic lordosis and sagittal balance and shows that we deal with very complex parameters that may or may not have a substantial effect on our patients.

Thus, it is unclear, how much correction is advantageous or even possibly harmful. It is still not clear, what impact radiological parameters have for the daily practice of a spine surgeon, for example: to plan an extensive surgery for a monosegmental pathology in order to meet the requirements of radiological parameters instead of going solely for the monosegmental pathology? The available evidence is controversial. That is why we did not just want to perform an analysis regarding the baseline criteria. We divided into subgroups with the question whether a special range of correction or a special terminal angle is superior to influence the clinical course.

We analyzed data of more than 100 patients suffering from CSM to assess the impact and validity of lordosis on the clinical outcome, especially compared to well-known and well-validated clinical parameters assessing CSM: the modified Japanese Orthopaedic Association scoring system (mJOAS) and the visual analogue scale for pain (VAS).

## Materials and methods

### Patient cohort

The authors analyzed the records of 102 patients who underwent cervical decompression surgery and instrumentation, completed at least a 1 year follow-up and had preoperative X-rays. The clinical data was prospectively collected in our department from a database maintained for quality control.

All patients suffered from CSM that was resistant to conservative treatment. A correlating spinal canal stenosis was confirmed by MRI or myelography in all cases.

An ethical vote was not required by our local institutions (Ethical Board of the Ärztekammer Berlin) for this retrospective data analysis as long as the patient’s anonymity is ensured (S1 File).

### Surgery

Our study pool comprised of patients with CSM that underwent surgery and instrumentation of the cervical spine with the aim to decompress the spinal cord and maintain or restore cervical lordosis. The indication for anterior or posterior single-level or multi-level surgery depended on several factors: extent and direction of spinal cord compression, extent of the spinal cord’s signal alteration in the MRI, segmental and cervical alignment. The indication for a certain surgical technique did not follow a fixed protocol, but the surgical approach was selected at the discretion of the operating surgeon.

The surgical techniques involved:

- Anterior cervical corpectomy and fusion (ACCF) (n = 36),
- Single- level anterior cervical discectomy and fusion (sACDF) (n = 24),
- Multi-level anterior cervical discectomy and fusion (mACDF) (n = 17),
- Laminectomy and fusion with lateral mass instrumentation (LF) (n = 25).

After surgery all patients were treated with the same protocol, which consisted of physical rest for 6–12 weeks and then physical therapy. A cervical collar was not applied.

### Clinical and radiological evaluation

For quality control purposes patients undergoing surgery in our institutions complete a questionnaire to assess the clinical condition before and after surgery. Follow-up examinations were performed on an outpatient basis. In all cases the clinical situation was assessed by evaluation of pre- and postoperative mJOAS [27], by visual analogue scale (VAS) for neck pain before and after surgery and postoperative Odom’s score [28].

Radiographic examinations included plain and functional radiography. Radiological analysis took the measurement of cervical lordosis between C2 and C7 according to Cobb. Measurements were done on digital radiographs using integrated software to measure angles up to an accuracy of 0.1° (AGFA Impax FX, Mortsel, Belgium). To validate the collected data the measurements were performed independently by two examiners.

The patients were separated into 3 groups to compare different amounts of surgical correction of the sagittal profile: Delta 1 (<0°), Delta 2 (1–7°), Delta 3 (≥8°).

We also divided the patients into different groups to assess the influence of the final lordosis on clinical outcome: Omega 1 (0–7°), Omega 2 (8–14°), Omega 3 (15–21°), Omega 4 (≥22°).

### Statistical analysis

The statistical evaluation was performed using IBM SPSS Statistics 22 and involved the T-test, Mann-Whitney U-test, variance analysis, Kruskal Wallis´ ANOVA, Wilcoxon signed-rank test and Kolmogorov-Smirnov test. A result with a p-value < 0.05 was considered to be significant.

## Results

### Demographic data

The average follow-up time was 85.48±56.05 months after surgery.

The age at surgery ranged from 36 to 85 years with a mean of 62.61±9.18 years with no difference between the groups (p = 0.423).

57 patients were male and 45 female. Gender had no influence on clinical outcome (for mJOAS p = 0.480, for VAS neck pain p = 0.613, for Odom’s score p = 0.239). Neither did the gender groups differ on these criteria before surgery (p = 0.257 for mJOAS and p = 0.950 for VAS neck pain).

### Clinical and radiological analysis

219 levels were fused in 102 patients (Table 1). 84 patients showed a preoperative lordosis, while 18 patients had a kyphosis. The statistical analysis of the two groups regarding surgical method showed no statistical difference (p = 0.190). Only patients who completed the regular 1-year-follow-up were included. Postoperative X-rays were available in 86 cases shortly after surgery, in 67 cases after 1 year and in 52 cases after more than 2 years (S2 File).

**Table 1:** Surgical technique stratified by preoperative alignment.

Lordosis (cobb angle pre-OP ≥ 0°)	n = 84
ACCF	n = 27
sACDF	n = 21
mACDF	n = 12
LF	n = 24
**Kyphosis** (cobb angle pre-OP < 0°)	**n = 18**
ACCF	n = 9
sACDF	n = 3
mACDF	n = 5
LF	n = 1

**Fig 1:**
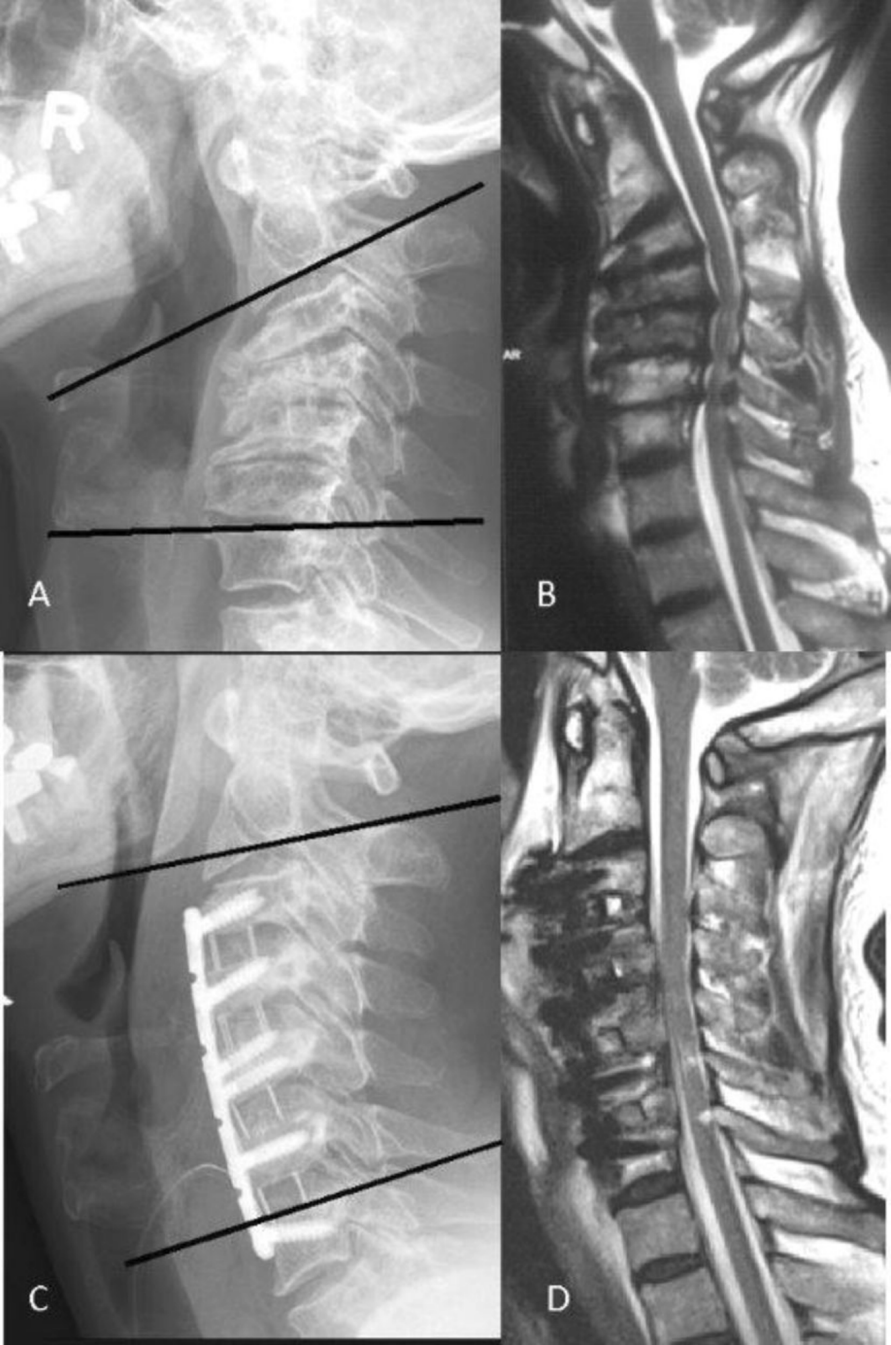
Preoperative radiograph (A) and MRI (B) of a patient suffering from CSM with a kyphotic sagittal alignment of -25°. The multilevel ACDF and plating restored a lordotic profile (+5°), but still by far does not reach physiological lordosis (C). We refer to these patients postoperatively as high Delta, low Omega. While at levels C4-7 a direct decompression by discectomy was performed, the level C3/4 shows an intact posterior longitudinal ligament and thus a good indirect decompression which was achieved solely by correcting the segmental kyphosis (D).

**Fig 2:**
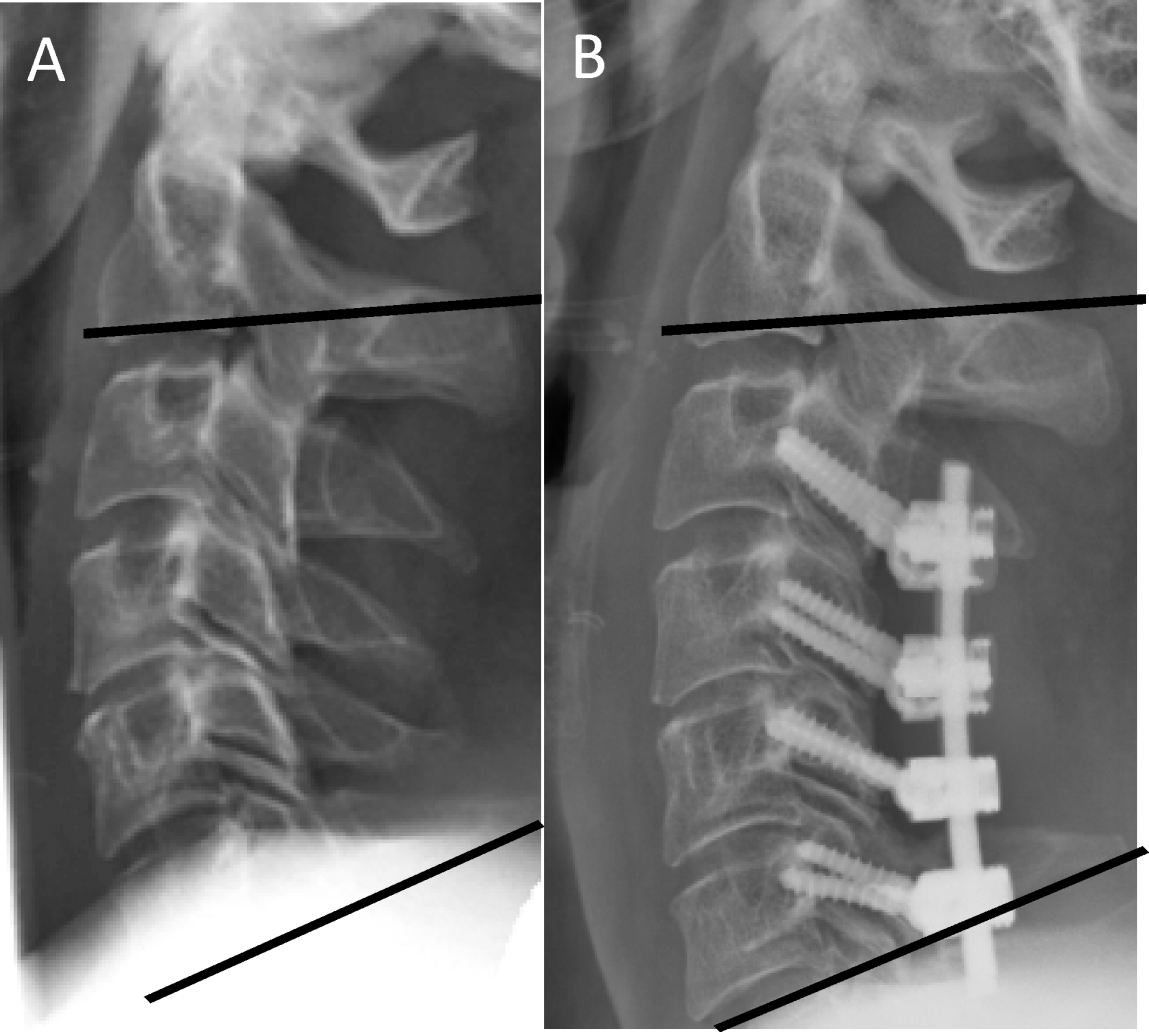
Pre- (A) and postoperative (B) radiographs of a patient suffering from CSM with a near-physiological lordosis of 20° that was largely maintained (referred to postoperatively as low Delta, high Omega).

The patients were evenly distributed for statistical comparison (Table 2). 75% of all patients considered the surgical treatment a success (excellent or good outcome). Surgery improved the neurological outcome according to mJOAS and neck pain significantly (Table 3). The clinical outcome was independent of pre- and post-operative cervical alignment, although the severity of radiological changes might have suggested substantial differences (Figs 1 and 2).

**Table 2:** Radiological data of changed cervical sagittal alignment (Delta groups).

Delta Group	I	II	III	
**Definition**	<0°	1–7°	≥8°	
**n**	38	38	26	Σ 102
**No. of instrumented levels (n)**	88	70	60	
**Instrumented levels/ patient**	2.32	1.84*	2.35	p = 0.045
**C2/7 pre**	21.3±8.43	11.5±7.49	-6.96±14.68	p<0.001
**C2/7 post**	16.29±8.68**	15.43±7.98**	13.71±10.90**	p = 0.497
**C2/7 last**	14.42±8.69	13.70±7.99	13.41±9.27	p = 0.885

Pre- and postoperative radiological data of patients divided into three different groups depending on amount of change (Delta) of the sagittal profile. Data given as mean with standard deviation before surgery (C2/7 pre), a few days after surgery (C2/7 post) and at last follow-up (C2/7 last).

**p<0.001

*p<0.05.

**Table 3:** Clinical data compared to sagittal change (Delta).

Delta Group	I			II	III	
**Definition**	<0°			1–7°	≥8°	
**n**	38			38	26	Σ 102
**VAS pre**	4.27±1.24			4.35±1.45	4.84±1.46	p = 0.257
**VAS last**	3.35±1.36**			3.16±1.69**	3.58±1.92*	p = 0.563
**mJOAS pre**	12.43±2.57			12.14±2.72	12.65±2.31	P = 0.815
**mJOAS last**	14.45±X2.45**			14.30±2.60**	14.73±1.95**	p = 0.908
**Odom´s Score**						p = 0.486
Excellent (n)		15	10		12	Σ 37
Good (n)		13	18		8	Σ 39
Fair (n)		8	9		5	Σ 22
Poor (n)		2	1		1	Σ 4
**Type of surgery (n)**						
sACDF		9	13		2	Σ 24
mACDF		3	5		9	Σ 17
ACCF		10	12		14	Σ 36
LF		16	8		1	Σ 25

Pre- and postoperative clinical data of patients divided into three different groups depending on amount of change of the sagittal profile (Delta). Data given as mean with standard deviation before surgery (“pre”) and at last follow-up (“last”). VAS = visual analogue scale of pain, mJOAS = modified Japanese Orthopaedic Association score, sACDF = single-level anterior cervical discectomy and fusion, mACDF = multilevel anterior cervical discectomy and fusion, ACCF = anterior cervical corpectomy and fusion, LF: laminectomy and fusion with lateral mass screws.

**p<0.001

*p<0.05.

### Change of cervical alignment (Delta-status)

All groups presented lordosis at final follow-up: The 26 Delta III patients were significantly corrected, Delta II-patients maintained lordosis or received only a moderate correction, and Delta I-patients even lost a certain degree of lordosis as even if they still displayed a lordosis of more than 14° at final follow-up (Table 2). Even patients with a significant loss of a preoperative large lordosis were shown to substantially profit from surgery with no differences in improvement compared to the other groups (Table 3, Delta I group). The subgroup analysis showed that less levels were instrumented in Delta-group II compared with Delta group III (p = 0.045). We compared patients with a low preoperative lordosis that featured a small and large amount of correction after surgery (low versus high delta) to assess the impact of correction in lordotic patients. We detected no statistical difference (p = 0.086). The comparison of Odom’s criteria showed that preoperatively kyphotic patients (n = 18) benefitted more from surgery than lordotic patients (n = 84) (p = 0.029). No difference was seen regarding mJOAS and VAS of neck pain comparing these two groups.

The Delta-I-group was made up of a large number of patients undergoing laminectomy and instrumentation (16 of 38 patients, Table 3).

Regardless of the pre- or postoperative cervical alignment surgery significantly improved neck pain and neurological status of all patients. Overall satisfaction was not connected to preoperative or postoperative cervical alignment (Table 3).

The interobserver variability measuring lordosis on radiographs did not affect the statistical analysis.

### Final lordosis (Omega-status)

A preoperative kyphosis was found in 18 of all 102 patients (17,65%). After surgical treatment a cervical lordosis was found in all 102 patients at the last follow up. Patients with a straight alignment of the cervical spine did not report more neck pain or present an inferior neurological outcome according to mJOAS compared to patients with a larger final lordosis (>15°, n = 53 patients) (Table 4).

**Table 4:** Clinical data in context with final sagittal alignment (Omega).

Omega Group	I	II	III	IV	
**Definition**	0–7°	8–14°	15–21°	≥22°	
**n**	26	24	32	20	Σ 102
**VAS pre**	4.67±1.34	4.30±1.36	4.19±1.36	4.74±1.52	p = 0.378
**VAS last**	3.27±1.73*	2.92±1.82*	3.52±1.52*	3.65±1.42*	p = 0.337
**mJOAS pre**	12.58±2.47	12.48±2.71	12.50±2.71	11.79±2.28	p = 0.646
**mJOAS last**	14.50±2.78 **	14.46±2.32**	14.59±2.28**	14.21±2.15**	p = 0.892
**Odom´s Score**					p = 0.260
Excellent (n)	11	5	11	10	Σ 37
Good (n)	8	11	13	7	Σ 39
Fair (n)	6	7	7	2	Σ 22
Poor (n)	1	1	1	1	Σ 4

Pre- and postoperative clinical data of patients divided into three different groups depending on their final sagittal profile (Omega). Data given as mean with standard deviation before surgery (“pre”) and at last follow-up (“last”). VAS = visual analogue scale of pain, mJOAS = modified Japanese Orthopaedic Association score.

**p<0.001

*p<0.05.

## Discussion

We present a retrospective study with prospectively acquired data of 102 patients and could show that decompression, stability and a final lordotic cervical alignment represent key factors for successful surgery, regardless of the exact amount of final cervical lordosis or achieved correction. Although a certain amount of neck pain and neurological impairment persists even after successful surgery patients that have lost lordosis through surgery or only gained a low amount of cervical lordosis profit as much as patients that maintain lordosis or achieve correction into a “physiological” cervical alignment.

### Sagittal alignment, decompression and stability

The loss of lordosis and the development of a kyphosis can be associated with neurological deterioration and should be avoided [24, 29–31]. However, even patients with a physiological cervical alignment and adequate decompression can display certain amounts of persisting neck pain and neurological dysfunction, as evidenced even in patients undergoing total disc arthroplasty that display only slight degenerative changes [32]. Kyphotic patients can be asymptomatic, as well [26]. This complicates the terminology of a “physiological lordosis” and “balance” even more.

The persistence of pain and subnormal mJOAS despite successful surgery and significant clinical improvement are probably results of the degenerative nature of a spondylotic disease and a limited recovery capacity of the impaired spinal cord. Most of our patients had a good recovery, an indicator that good preoperative conditions may lead to favorable results [33, 34]. But patients with a low baseline mJOAS, although they do not reach near-normal values anymore, benefit from surgery, nonetheless. This is consistent with findings of other studies [8, 35].

The loss of lordosis especially in the Delta I-group can be explained by cage subsidence, problems with the positioning of the head for a posterior approach and the choice of implant [2]. In case of a large lordosis smaller alterations might not be recognized during the positioning of the head, thus they should be anticipated and controlled even more precisely.

Patients’ baseline neck pain and mJOAS did not differ between the groups regardless of preoperative cervical alignment. This goes conform with other studies [22]. Due to different reasons (cage subsidence, loss of lordosis due to surgical technique) all of the delta groups ended up at similar mean one-year Cobb angles, and may be why there does not appear to be a difference comparing clinical parameter. However, the comparison of the Omega groups showed that different final Cobb angles may result in similar outcomes. Our kyphotic patients benefitted more from surgery than preoperatively lordotic patients regarding Odom’s criteria, but the comparison is hindered by different numbers of patients (18 versus 84).

Instead of measuring the local SVA we chose to determine the well-established Cobb-angle to assess kyphosis and lordosis and compare the sagittal angle with the clinical outcome. While SVA-parameters of less than 15mm may be more often observed in asymptomatic patients [17], the patient in Fig 1 displays a severe kyphosis before surgery, but does not display a displacement of the C2 plumb line to the front. However, this is a singular, non-representative case, and we did not assess the SVA parameter in our patients. Therefore we believe that the determination of kyphosis/lordosis and the appraisal of its influence on the clinical outcome in CSM-patients might be even more complex than currently anticipated. This could potentially have ramifications for the planning of surgeries. An enormous correction of the spinal profile into “physiological” ranges might bear disadvantages, such as the risk of C5 palsy resulting from the shift of the spinal cord. About 20° of correction appear to represent a critical crossing line [25]. There is an increasing scope of different radiological measurement methods that may have a substantial impact on the clinical course. These parameter often strongly correlate. Maybe one method alone will be regarded as the “gold standard” in the future. We could only provide the long-time established C2-7 Cobb angle in our database. Providing all of these parameters (T1-slope, C2-7 SVA, C2-7 Cobb angle) and to compare them with the clinical outcome would have been highly interesting and desirable of course.

Decompression, stability and lordosis appear to be key factors for a successful surgical treatment of CSM. All of our patients were decompressed and stabilized and had reached lordosis at final follow-up. Nonetheless, an effective decompression can be achieved by a laminoplasty and thus mobility preserved as long as the lordosis of the cervical spine is maintained; a laminoplasty is not advisable in cases of a pre-existing kyphosis [36, 37]. So the avoidance of a destabilization and development of a kyphosis are mandatory. The exact amount of lordosis seems to play a very complex role, as our findings show that all of our patients profited from surgery no matter which group: patients with a straight alignment as well as patients with a large lordosis. This might be attributed to adopted muscular patterns and body positioning to compensate the changes and effects of the slowly developing degenerative disease. Comparing cervical alignment, neurologic function, pain and quality of life showed that a lordosis of at least 6° can mean a relevant difference for neck pain [12]. However, 6° remains still a value that is far from a near-physiological lordotic profile.

### Surgical technique

When observing the neurological recovery none of the various surgical approaches offered consistent advantages over the others [2, 38–40], though the various procedures show differing results regarding spinal canal increase, bleeding, fusion rate and surgical complications [41–43]. Anterior compression that involves 1 or 2 vertebral bodies is usually addressed by an anterior approach [44, 45], while in cases of more than 2 levels a posterior procedure might be more suitable to avoid difficulties while swallowing and implant failure [44–47]. Addressing multiple levels helps to achieve a better correction as resembled in our subgroup analysis. Our Delta III group involved more instrumented levels than Delta II. In cases of multilevel disease with kyphosis, a combined approach may be of advantage to reconstruct the sagittal plane and decompress from posterior [48]. The surgeons of our patients adhered to these principles, but did not follow a fixed protocol.

### Limitations of the study

The allocation to certain groups was performed after collection and analysis of patient data to perform a statistical analysis of groups of almost similar size. This might represent an analytical bias, but there are no scientific criteria to distinguish lordotic profiles.

The discussion of the impact of regional alignment on outcomes is strongly limited by the lack of evaluation of more global alignment. The required degree of cervical lordosis post-operatively appears to be closely linked to the slope of the T1 segment, which is widely accepted as a marker for other global parameters [49, 50], but many compensatory changes occur that might lead to asymptomatic conditions [51]. We have not measured the T1-slope in our patients. Nonetheless, the impact of global spine parameters and the T1-slope are still under discussion and are not validated by level 1 studies, although prospective multicenter data already exist [52]. Many factors influence the outcome above all of these parameters [53]. Therefore it is still not clear what impact these radiological parameters have for the daily practice of a spine surgeon. For example: should a surgeon plan an extensive surgery for a monosegmental pathology in order to meet the requirements of global parameters instead of going for the monosegmental approach that offers good clinical outcomes regardless of these complex parameters?

Given that variability in post-operative alignment is minimal and the lack of kyphotic patients following surgery the conclusions that decompression and instrumentation represent main factors for positive outcome might be difficult. It does not support the conclusion that sagittal alignment should not be addressed. The lack of more functional scores, such as neck disability index (NDI) and, even more important, a quality of life (QOL) score, represents another limitation of the study.

The surgeries were performed by different spine surgeons which represents a potential bias of the study regarding personal preferences, indication for surgery etc. The approach was not standardized.

The retrospective study design represents another limitation of the study. I.e. we did not analyze the impact of duration of symptoms as the data provided from the records was not specific enough. It is not a consecutive series. The range in time of the follow-up examinations provides a larger possibility of interpretation when compared to prospective studies. The indication for single-level or multi-level surgery did not depend solely on the extent of the spinal cord compression, but also on the cervical alignment: a considerable segmental kyphosis was corrected by a multi-level anterior approach. The indication for anterior or posterior surgery depended on various factors as well: direction of spinal cord compression and preoperative kyphosis. Therefore only the posterior group offered patients with a preoperative lordosis.

The improvement of pain and neurological impairment supports the statistical impact of the data and their validity despite comparatively small numbers of patients and many limitations of the study.

## Conclusion

Decompression of the spinal cord, stability and restoration or preservation of lordosis appear to be important for the successful treatment of CSM. While the achievement of cervical lordosis remains a major goal of surgery, clinical improvement is not hindered in patients with a small lordosis. Kyphosis should be eliminated in symptomatic patients. Patients with a large correction of a kyphosis but small final lordosis have similar outcomes as patients with physiological lordotic profiles. Persisting pain might be attributed to the degenerative nature of the underlying disease. The individual optimal sagittal alignment in patients suffering from CSM might differ substantially from healthy persons and appears to be even more complex to be determined than anticipated.

## Supporting information

S1 File The correspondence with the ethical committee and translation. (PDF)

S2 File The anonymised data. (XLS)
