# Supplementary material for: The influence of sagittal profile alteration and final lordosis on the clinical outcome of cervical spondylotic myelopathy. A Delta-Omega-analysis
Source: PLoS One. 2017 Apr 21;12(4):e0174527. doi: 10.1371/journal.pone.0174527 (PMC5400234; doi:10.1371/journal.pone.0174527)
Supplement: S1 File — (PDF) [file pone.0174527.s001.pdf]

ETHIK-KOMMISSION

ÄRZTEKAMMER BERLIN

ÄRZTEKAMMER BERLIN Friedrichstraße 16 · 10969 Berlin

Vivantes
Auguste-Viktoria-Klinikum
Herrn PD Dr. med. Mario Cabraja
Rubensstr. 125
12157 Berlin

Körperschaft des
öffentlichen Rechts

Ansprechpartner Carola Jasper

Telefon 0 30 / 4 08 06 - 2646
Zentrale 0 30 / 4 08 06 - 0
Fax 0 30 / 4 08 06 - 2299

Email ek@aekb.de
nicht für Dokumente mit elektronischer Signatur

www. aerztekammer-berlin.de

Berlin, 23. Dezember 2016

Ihre Anfrage vom 16.12.2016
Ihr Vorhaben: „Retrospektive Analyse von Daten aus der klinischen Routine zum Einfluss des postoperativen sagittalen Profils und Alignementkorrektur auf das klinische Outcome bei zervikaler spondylotischer Myelopathie"

Sehr geehrter Herr Dr. Cabraja,

anbei übersenden wir Ihnen die Stellungnahme des Vorsitzenden unserer Ethik-Kommission zu Ihrer Anfrage vom 16.12.16:

> *Eine retrospektive Aktenauswertung unterliegt nicht der Beratungspflicht durch die Ethik-Kommission der Ärztekammer Berlin, soweit die Daten anonymisiert sind.*
> *gez.*
> *PD Dr .med. Hans-Herbert Fülle*
> *Vorsitzender der Ethik-Kommission*
> *der Ärztekammer Berlin*

Mit freundlichen Grüßen

Carola Jasper
Abteilung 3,
Ethik-Kommission

Translation

Your inquiry from 16.12.2016

Your study: Retrospective analysis of data from the clinical routine on the impact of postoperative sagittal profile and correcture of alignment on the clinical outcome of cervical spondylotic myelopathy.

Dear Dr. Cabraja,

We send you the statement of the chairman of our ethics commitee to answer your request from 16th December 2016:

> *A retrospective data analysis does not fall under the obligation for counsel by the ethics committee of the Ärztekammer Berlin as long as the data is anonymous.*
>
> *Signed on behalf of*
>
> *Associate professor Dr. Hans-Herbert Fülle*
>
> *Chairman of the ethics commitee of the Ärztekammer Berlin*

With kind regards

Carola Jasper

Department 3

Ethics commitee
